# Development of genomic predictions for harvest and carcass weight in channel catfish

**DOI:** 10.1186/s12711-018-0435-5

**Published:** 2018-12-14

**Authors:** Andre L. S. Garcia, Brian Bosworth, Geoffrey Waldbieser, Ignacy Misztal, Shogo Tsuruta, Daniela A. L. Lourenco

**Affiliations:** 1 Animal and Dairy Science Department, University of Georgia, Athens, GA 30602 USA; 2 Warmwater Aquaculture Research Unit (WARU), USDA-ARS, Stoneville, MS 30776 USA

## Abstract

**Background:**

Catfish farming is the largest segment of US aquaculture and research is ongoing to improve production efficiency, including genetic selection programs to improve economically important traits. The objectives of this study were to investigate the use of genomic selection to improve breeding value accuracy and to identify major single nucleotide polymorphisms (SNPs) associated with harvest weight and residual carcass weight in a channel catfish population. Phenotypes were available for harvest weight (n = 27,160) and residual carcass weight (n = 6020), and 36,365 pedigree records were available. After quality control, genotypes for 54,837 SNPs were available for 2911 fish. Estimated breeding values (EBV) were obtained with traditional pedigree-based best linear unbiased prediction (BLUP) and genomic (G)EBV were estimated with single-step genomic BLUP (ssGBLUP). EBV and GEBV prediction accuracies were evaluated using different validation strategies. The ability to predict future performance was calculated as the correlation between EBV or GEBV and adjusted phenotypes.

**Results:**

Compared to the pedigree BLUP, ssGBLUP increased predictive ability up to 28% and 36% for harvest weight and residual carcass weight, respectively; and GEBV were superior to EBV for all validation strategies tested. Breeding value inflation was assessed as the regression coefficient of adjusted phenotypes on breeding values, and the results indicated that genomic information reduced breeding value inflation. Genome-wide association studies based on windows of 20 adjacent SNPs indicated that both harvest weight and residual carcass weight have a polygenic architecture with no major SNPs (the largest SNPs explained 0.96 and 1.19% of the additive genetic variation for harvest weight and residual carcass weight respectively).

**Conclusions:**

Genomic evaluation improves the ability to predict future performance relative to traditional BLUP and will allow more accurate identification of genetically superior individuals within catfish families.

## Background

Catfish farming is the largest aquaculture segment in the US, accounting for approximately 50% of US food-fish production [1]. The US catfish industry is based on the production of channel catfish (*Ictalurus punctatus*) and the hybrid between the channel and blue catfish (*Ictalurus furcatus*). To provide a centralized source for US catfish production research, the USDA-ARS Warmwater Aquaculture Research Unit (WARU) was established in Stoneville, MS. As part of its mission to improve catfish production efficiency, the WARU has conducted a channel catfish breeding program since 2006, primarily selecting fish for increased growth and carcass yield.

Traditional evaluation using pedigree-based best linear unbiased prediction (BLUP) has been applied since the beginning of the breeding program at WARU. To investigate the potential for implementing genomic selection in the WARU catfish breeding program, animals were genotyped using a 57 K single nucleotide polymorphism (SNP) array. Dense markers are used as an extra source of information to estimate breeding values [2] in breeding programs for several livestock species because of the potential increase in accuracy of estimated breeding values (EBV). Another advantage of genomic selection, which is particularly important to aquaculture breeding, is the ability to exploit within-family genetic variation for animals that do not have records [3].

One of the methods available for genomic evaluation is single-step genomic BLUP (ssGBLUP) [4]. This method combines phenotypes, pedigree, and genotypes, and potentially gives more accurate and less biased genomic EBV (GEBV) than multistep methods [5]. In ssGBLUP, the relationship matrix is a combination of pedigree and genomic relationships [4, 6]; therefore, information on all animals can be used in the evaluation, regardless of genotyping status.

The accuracy of genomic evaluation depends on several factors including linkage disequilibrium (LD) between markers and quantitative trait loci (QTL), effective population size ($N_{e}$), and the relationship among individuals in training and validation data [7, 8]. Thus, investigating the $N_{e}$ and the extent of LD can give clues about how much genetic gain can be obtained by adopting genomic selection, how many animals should be genotyped, and potentially, how many SNPs should be included in the marker panel. The possibilities of using lower density SNP chips to reduce costs and promote adoption of genomic selection, and searching for individual SNPs explaining major portions of variance should also be explored. If major SNPs explain a reasonable proportion of the genetic variance observed for a trait, selection based on a limited number of SNPs can be performed.

The first objective of this study was to investigate the feasibility of implementing genomic evaluation in US channel catfish by using ssGBLUP. The second objective was to determine the presence of potential regions in the genome that contain SNPs with major effects on harvest weight and residual carcass weight (i.e. carcass weight adjusted for harvest weight).

## Methods

### Data

Data from the USDA-ARS Warmwater Aquaculture Research Unit (WARU) were available for this study. Harvest weight and carcass weight (i.e., the weight of a fish with intact skin, but removed head and viscera) were recorded from 2008 to 2015, with a total of 27,160 and 6020 records, respectively, and pedigree information was available for 36,365 fish. Among those, 27,883 had either phenotypes/genotypes or were related to phenotyped/genotyped fish. This population constitutes the Delta Select strain that was developed based on 10 to 13 egg-masses collected from eight commercial catfish farms in the spring of 2006 (total = 97 egg masses). Each egg-mass was assumed to be a single full-sib family and families were assumed to be unrelated to each other. Each egg-mass was hatched in a separate hatching tank, fry were reared in separate full-sib family tanks until the fingerling stage when ~ 50 fish per family were tagged with passive integrated transponders (PIT tags) and stocked communally in earthen ponds where they were grown until the fall of 2007. At harvest, gender and weight of all fish were recorded, and an average of seven males and six females were randomly selected from each full-sib family and kept as broodfish. In addition to these fish, mature fish were obtained from two additional farms (40 males and 39 females from one farm, and 20 males and 59 females from the other farm). The broodfish from the base population were allowed to mate at random until 2 and 3 years old, and offspring represent the 2008 and 2009 year-class. Parentage was determined by genotyping fish for 16 microsatellites [9]. In total, 181 and 198 families were produced in 2008 and 2009, respectively. The families were reared separately until tagging (about 280 days old). Approximately 30 fish per family were tagged and reared communally in earthen pounds. Harvest weight was recorded when the animals were about 16 months old and a month later, approximately seven fish per family were processed for carcass weight recording.

Variance components and EBV were estimated and broodfish were selected using an index, which was the average standardized EBV for harvest weight and residual carcass weight. This approach was used to equalize selection emphasis on each trait. The fish selected from the 2008 and 2009 year-class (first generation of selection) were spawned in ponds in 2011 and 2012 as 2-, 3- and 4-year old fish. Performances of the 2011 and 2012 year-class progeny reflect effects of one generation of selection. Progeny from the 2011 and 2012 year-classes were evaluated and selected on the same index, spawned in ponds in 2014 and 2015 as 2-, 3- and 4-year old. Progeny from the 2014 and 2015 year-class were evaluated as described previously, and their performance reflects effects of the second generation of selection. Approximately 10% of the harvested fish from each year-class were kept as broodfish and no more than 10% of selected broodfish were from a single full-sib family to limit inbreeding. From 110 to 198 full-sib families were evaluated for each year-class and 954 and 752 full-sib families were evaluated for harvest weight and residual carcass weight, respectively.

Broodfish were stocked in March of each spawning year into 0.04 to 0.1 ha earthen ponds at a rate of 800 to 1000 kg per ha and stockings were designed to prevent mating among full-sibs. Male to female ratios in brood ponds ranged from 1:1 to 1:2. In early April, weighted plastic ‘spawning-cans’ were placed in ponds to provide spawning sites, and cans were inspected for the presence of egg-masses two or three times a week. Egg-masses were collected from ponds and transported to the hatchery. Fry were reared in separate full-sib tanks until the fingerling stage at which point they were tagged and stocked communally in earthen ponds and fed daily. Appropriate commercial catfish diets were provided and proper water quality was maintained throughout the year.

Genomic DNA from 49 founders of the Delta Select strain (described above) was sequenced with 2 × 150 bp reads on the NextSeq 500 platform (Illumina Inc., San Diego, CA) to obtain approximately 5× genome coverage per individual (25–40 million read pairs per individual). Paired sequences were aligned to the reference genome [10] using BWA-MEM [11] and variants were identified using the Genome Analysis ToolKit [12]. The GATK best practices workflow was used to identify SNPs and indels in individuals (HaplotypeCaller) and then jointly across the population (GenotypeGVCFs). The analysis produced more than 15 million raw variants (SNPs plus indels) and more than 12 million raw SNPs. Filtering for strand bias, map quality, and depth of coverage ($\le$ mean + 2 standard deviations) reduced the number of high-quality putative SNPs to 7,445,905. Further filtration to identify SNPs that were positioned at least 50 bp from another SNP or indel and with a minor allele frequency higher than 0.05 reduced the number of candidate SNPs to 1,661,221.

An Axiom custom screening array (ThermoFisher Scientific, Waltham, MA) was produced using 660,000 SNPs, and 162 channel catfish were genotyped to validate the selected SNPs. Six doubled haploid (homozygous) catfish were also included to identify false heterozygosity at loci within genomic repeats. A total of 489,390 loci were called as polymorphic, high resolution loci on the array, and 340,737 loci were uniquely located on the catfish genome assembly. After the removal of 17,635 loci that demonstrated heterozygosity in the doubled haploids, 323,102 converted SNPs were available. A custom python script (Guangtu Gao, personal communication) was used to select SNPs that were evenly distributed across each of the 29 chromosomes. A new custom Axiom genotyping array was produced, which contained 57,354 SNPs with an average distance between markers of 13.3 kb. The final genotype data included 2911 animals, each genotyped at 54,837 SNPs after quality control. The SNPs excluded in the quality control had a minor allele frequency lower than 0.05, were monomorphic or had a call rate lower than 90%. Genotyped animals were excluded if the call rate was lower than 90% (i.e., 10% of the genotypes were missing). Among the animals that passed the quality control, 2826 had records on harvest weight and 969 on carcass weight. The distribution of genotypes and phenotypes based on year-class is in Table 1.

**Table 1:** Distribution of phenotypes and genotypes by year-class

Year-class	Full-sib families	Harvest weight	Carcass weight	Genotyped animals
Before 2006	–	–	–	70
2006	–	–	–	2
2008	181	4762	829	78
2009	198	5686	1352	44
2011	180	1982	–	38
2012	110	4484	924	133
2014	113	4141	955	189
2015	172	6105	1960	2357
Total	954	27,160	6020	2911

### Model and analysis

Single-trait animal models were used for harvest weight and residual carcass weight. For harvest weight, the model was:

1 $${\mathbf{y}}_{{\mathbf{w}}} = {\mathbf{Xb}} + {\mathbf{Zu}} + {\mathbf{Wp}} + {\mathbf{e}},$$

where ${\mathbf{y}}_{{\mathbf{w}}}$ is a vector of harvest weight; ${\mathbf{b}}$ is a vector of fixed effect of year-sex-pond interaction, and age (ranging from 391 to 620 days) as a linear covariable nested within sex; ${\mathbf{u}}$ is a vector of additive direct genetic effect; ${\mathbf{p}}$ is a vector of common environmental effect, which accounts for the fact that full-sibs from the same spawn were raised in the same tank until they reach an age and weight suitable for tagging (average tagging weight of 119.3 g and average tagging age of 271 days); ${\mathbf{e}}$ is the vector of residuals; ${\mathbf{X}}$, ${\mathbf{Z}}$, and ${\mathbf{W}}$ are incidence matrices for the effects contained in ${\mathbf{b}}$, ${\mathbf{u}}$, and ${\mathbf{p}}$, respectively.

For residual carcass weight, the model was:

2 $${\mathbf{y}}_{{\mathbf{c}}} = {\mathbf{X}}_{1} {\mathbf{b}}_{1} + {\mathbf{X}}_{2} {\mathbf{b}}_{2} + {\mathbf{Zu}} + {\mathbf{Wp}} + {\mathbf{e}},$$

where ${\mathbf{y}}_{{\mathbf{c}}}$ is a vector of carcass weight; ${\mathbf{b}}_{1}$ is a vector of linear covariables for body weight nested within year-sex interaction; ${\mathbf{b}}_{2}$ is a vector of fixed effect of year-sex-pond interaction; ${\mathbf{u}}$, ${\mathbf{p}}$, and ${\mathbf{e}}$ are described above; ${\mathbf{X}}_{1}$ and ${\mathbf{X}}_{2}$ are incidence matrices for the effects contained in ${\mathbf{b}}_{1}$ and ${\mathbf{b}}_{2}$. The term residual carcass weight arose from the fact that adjusting carcass weight to a common body weight allows identification of fish that have a higher proportion of whole weight as saleable carcass. The idea is similar to the residual feed intake which is widely used in livestock breeding.

Traditional BLUP and ssGBLUP analyses were performed using the BLUPF90 family of programs [13]. In the mixed model equations for ssGBLUP, the inverse of the pedigree relationship matrix (${\mathbf{A}}^{ - 1}$) is replaced by ${\mathbf{H}}^{ - 1}$ [4], the realized relationship matrix that combines pedigree and genomic relationships:

3 $${\mathbf{H}}^{ - 1} = {\mathbf{A}}^{ - 1} + \left[ {\begin{array}{*{20}c} 0 & 0 \\ 0 & {{\mathbf{G}}^{ - 1} - {\mathbf{A}}_{22}^{ - 1} } \\ \end{array} } \right],$$

where ${\mathbf{G}}^{ - 1}$ is the inverse of the genomic relationship matrix and ${\mathbf{A}}_{22}^{ - 1}$ is the inverse pedigree relationship matrix for genotyped animals. The ${\mathbf{G}}$ matrix was constructed as in VanRaden [14]:

4 $${\mathbf{G}}\,{ = }\,\frac{{{\mathbf{MDM}}^{\prime } }}{{ 2\sum {\text{p}}_{\text{j}} ( 1 -{\text{p}}_{\text{j}} )}},$$

where ${\mathbf{M}}$ is a matrix of genotypes centered by twice the current allele frequencies ($p$); ${\text{j}}$ is the ${\text{j}}$th locus; ${\mathbf{D}}$ is a diagonal matrix of SNP weights with a dimension equal to the number of SNPs. All SNPs were assumed to have homogeneous weights in ssGBLUP, meaning that ${\mathbf{D}}$ was an identity matrix (${\mathbf{I}}$). To avoid singularity problems, ${\mathbf{G}}$ was blended with 5% of ${\mathbf{A}}_{22}$.

### Validation

The main interest in fish breeding is to better predict genetic merit of a fish as broodstock; however, the data collected so far during this first development of genomic predictions for catfish in the US do not allow a comparison between mid-parent GEBV and progeny performance, but this comparison will soon be possible. In our study, most of the genotyped animals with phenotypes were from the same year-class (i.e., 2015), precluding the use of validation on progeny performance and also forward prediction (i.e., future performance on individual fish). Therefore, to compare predictive ability of traditional and genomic evaluations, we conducted validations using several strategies to split fish into training and validation datasets.

Strategies 1 and 2 were used for both harvest weight and residual carcass weight. Strategy 1 was a random *k*-*fold* cross-validation, where the dataset was randomly split into k folds, predicting one fold based on k-1 folds. Genotyped animals with phenotypes were randomly split into 5 or 10 mutually exclusive groups (k = 5 or k = 10, respectively). In each round of cross-validation, phenotypes from one group (i.e., validation group) were removed from the dataset and the remaining folds (i.e., training group) were used to predict the future performance for animals in the validation group. This k-folds cross-validation was replicated five times and results are presented as the mean and standard error for the five replicates. In the validation strategy 2, genotyped full-sibs were split into two groups with one group used for training and the other group used for validation, and all phenotypes of the validation group were removed from the evaluation. This scenario is most important when phenotypes are measured on sibs of the selection candidates.

Validation strategies 3 and 4 were conducted for residual carcass weight only to evaluate the importance of collecting genotypes on fish that will be slaughtered for phenotype recording. Carcass weight requires the slaughtering of many animals and thus their removal from the pool of selection candidates and is also considerably more expensive to measure than harvest weight. Harvest weight is quickly and inexpensively measured on all selection candidates and therefore, evaluating scenarios 3 and 4 for harvest weight provided no realistic benefit. Strategy 3 was similar to strategy 2 except that we assumed that only half of the full-sibs in the training population had phenotypes. This third validation strategy would be especially important for carcass traits to reduce the number of genotyped animals that are slaughtered to collect phenotypes. The validation group remained the same as in scenario 2.

In strategy 4, training animals had genotypes, but no phenotypes and the validation group remained the same. The ssGBLUP method uses all available information in the evaluation, meaning that phenotypes for 5051 ungenotyped, slaughtered fish were included. In this way, genotyped animals could benefit from phenotypes of ungenotyped animals if both groups are related through the pedigree relationship matrix although no genotyped animals had phenotypes for carcass weight. This scenario was proposed because the cost of genotyping fish can be as high as the value of a fish itself. If genotyped fish have to be slaughtered for phenotype recording and they are removed as selection candidates, the cost of implementation of genomic selection would likely increase.

Trait heritabilities with the full data were 0.27 and 0.34 for harvest weight and residual carcass weight, respectively. As we changed the data structure by creating different training datasets for each validation strategy, we also estimated updated variance components to evaluate how changing the animals used in the training set analysis (which also changed the subsequent variance components) impacted predictive ability and inflation of (G)EBV. Reverter et al. [15] pointed out that breeding value inflation or deflation can be introduced if variance components do not reflect the actual data.

Ability to predict performance was used to compare traditional and genomic models. It was calculated as the correlation between (G)EBV for validation animals and phenotypes adjusted for fixed effects ($$y^{*} )$$, as described in (1) and (2), which were estimated based on the full data:

5 $$predictive\, ability = cor\left[ {\left( {\text{G}} \right){\text{EBV}}, y^{*} } \right],$$

In addition, the regression coefficient ($b_{1}$) of adjusted phenotypes on (G)EBV was used as a measure of inflation of breeding values.

6 $$y^{*} = b_{0} + b_{1} \times \left( {\text{G}} \right){\text{EBV }} + {\text{e ,}}$$

A regression coefficient lower than 1 indicates (G)EBV inflation, whereas a value higher than 1 indicates deflation.

### Genome-wide association

A genome-wide association study (GWAS) was performed to identify possible regions of the genome containing SNPs with major effects on harvest weight or residual carcass weight. Weighted ssGBLUP (WssGBLUP; Wang et al. [16]) implemented in postGSf90 from the BLUPF90 family of programs [13] was used for the GWAS. In the first implementation of WssGBLUP, Wang et al. [16] suggested that SNP weights should be calculated as $d_{j} = \hat{a}_{j}^{2} 2p_{j} \left( {1 - p_{j} } \right)$, following the formula for genetic variance due to an additive locus [17]. However, Lourenco et al. [18] showed that this method did not reach convergence under a more polygenic scenario because of extreme weights. Therefore, the SNP weights used in this study were described by VanRaden [14] as non-linear A weights:

7 $$d_{j} = {\text{CT}}^{{\frac{{\left| {{\hat{\text{a}}}_{j} } \right|}}{{sd\left( {{\hat{\text{a}}}} \right)}} - 2}} ,$$

where ${\text{CT}}$ is a constant that determines the departure from normality; $\left| {\hat{a}_{j} } \right|$ is the absolute estimated SNP effect for marker $j$, and $sd\left( {\hat{a}} \right)$ is the standard deviation of the vector of estimated SNP effects. non-linear A weights had good convergence properties and avoided extreme values (Breno O. Fragomeni, personal communication). This is because the maximum change in weights is limited by the minimum between 5 and the exponent of ${\text{CT}}$. In our study, ${\text{CT}}$ received a value of 1.125 based on Legarra et al. [19] and VanRaden [14]. Although these values were empirically derived based on dairy cattle populations, they resulted from tests over several traits with a more polygenic architecture.

The WssGBLUP is an iterative process. Wang et al. [16] and Zhang et al. [20] suggested that two iterations of weights were sufficient to maximize genomic accuracy and to correctly identify major SNPs in WssGBLUP. Based on the non-linear A weights, the number of iterations to reach convergence may vary from 5 to 10 (Breno O. Fragomeni, personal communication). Therefore, we chose five iterations and checked the stability of predictive ability and regression coefficients of adjusted phenotypes on GEBV. Predictive ability and inflation can be used as indicators for convergence when computing SNP weights in WssGBLUP [16]. After investigating which iteration had the highest predictive ability, based on reduced data, WssGBLUP was applied to the full data for harvest weight and residual carcass weight, and Manhattan plots were drawn for that iteration.

Manhattan plots were drawn based on the proportion of additive genetic variance explained by windows of 20 adjacent SNPs. The concept of SNP windows is rather abstract and tries to approximate haplotype blocks; therefore, it assumes that windows may be inherited together, which may not always be the case for all assumed windows.

### Linkage disequilibrium and effective population size

We used the first medium density SNP array (55 K SNP) developed for channel catfish in this study. However, we also examined linkage disequilibrium (LD) to determine the feasibility of using a lower cost, reduced SNP panel for genomic selection in this population.

In our study, LD was calculated with preGSf90 using the following equation:

8 $$r^{2} = \frac{{D^{2} }}{{P_{A} P_{a} P_{B} P_{b} }},$$

where $D = P_{AB} - P_{A} P_{B}$; $P_{AB}$ is the frequency of the genotype $AB$; $P_{A}$, $P_{a}$, $P_{B}$ and $P_{b}$ are the allele frequencies. The LD was calculated as the average of adjacent SNPs within chromosomes and across the genome.

A curve that fits the LD decay with distance between markers for each chromosome was calculated by fitting the equation proposed by Sved [21]:

9 $$E\left[ {r_{t}^{2} } \right] = \frac{1}{{1 + 4Ne_{t} d_{ij} }},$$

where $d_{ij}$ is the distance between markers $i$ and $j$ in Morgan and $Ne_{t}$ is the effective population size for the chromosome $t$, calculated as proposed by Saura et al. [22]:

10 $$Ne_{t} = \left( {4d_{t} } \right)^{ - 1} \left[ {\left( {r_{t}^{2} - N^{ - 1} } \right)^{ - 1} - \alpha } \right],$$

with $d_{t}$ as the average chromosome length in Morgan; $r_{t}^{2}$ is the average LD at chromosome $t$; $N^{ - 1}$ is the adjustment term for sample size (number of genotyped animals); and $\alpha$ is a fixed parameter that is assumed to be 1 if mutation is not considered and 2 if it is considered; we considered $\alpha = 2$.

Besides chromosome-based $N_{e}$, we also calculated $N_{e}$ based on the rate of inbreeding by generation using the of formula Falconer et al. [17]:

11 $$Ne_{F} = \frac{1}{2\Delta F},$$

where

12 $$\Delta F = \frac{{F_{n} - F_{n - 1} }}{{1 - F_{n - 1} }},$$

with $F_{n}$ as the inbreeding coefficient in the $n$th generation.

## Results and discussion

### Predictive ability and inflation

Table 2 shows the predictive ability for both traits under different validation strategies. In all validations, using genomic information through ssGBLUP improved the ability to predict future fish performance relative to traditional BLUP.

**Table 2:** Predictive ability for harvest weight and residual carcass weight under BLUP and ssGBLUP for all validation scenarios

Validation strategy	Validation scenarios^a^	Harvest weight		Residual carcass weight	
		BLUP	ssGBLUP	BLUP	ssGBLUP
1	Five fold cross-validation^b^	0.29^0.001^	0.37^0.001^	0.24^0.002^	0.31^0.002^
1	Ten fold cross-validation^b^	0.29^0.0003^	0.37^0.0004^	0.24^0.002^	0.32^0.002^
2	Full sib validation	0.31	0.38	0.25	0.34
3	Half of the full sibs with phenotypes	–	–	0.23	0.28
4	No phenotypes for all genotyped animals	–	–	0.22	0.24

Predictive ability is measured by the correlation between (G)EBV and phenotypes adjusted for fixed effects in the validation population

^a^Updating variance components or not produced exactly the same predictive ability for all scenarios

^b^Average and standard error across five replicates

In general, cross-validation scenarios using either k = 5 or k = 10-fold scenarios had very similar predictive ability. In addition, updating the variance components for different training datasets did not affect predictive ability, as expected [15]. Including genomic information increased predictive ability by 28% (for both five and ten fold) for harvest weight, and by 29% and 33% (five and ten fold, respectively) for residual carcass weight relative to traditional BLUP.

Validation strategy 2 (splitting full sibs into training and validation sets) resulted in overall predictive abilities for traditional BLUP and ssGBLUP that were greater compared to k-fold cross-validations. This was likely due to closer relationships between animals in training and validation groups [23] in strategy 2. The ssGBLUP outperformed BLUP by 23% for harvest weight and by 36% for residual carcass weight in strategy 2. Genomic information may have more impact on traits that cannot be measured on the selection candidates [24], such as carcass and disease resistance traits. For instance, in our study the greatest increase in predictive ability was for residual carcass weight.

Validation strategy 3, where only a portion of the full-sibs in the training set had phenotypes, had a predictive ability slightly higher than strategy 4 (no phenotypes on genotyped animals), but lower than those for validation on full-sibs with genotypes and phenotypes (strategy 2) and k-folds cross-validation (strategy 1). The gain in predictive ability of GEBV over EBV in strategy 3 was 22% for residual carcass weight. The drop in predictive ability for residual carcass weight for strategy 3 relative to strategies 1 and 2 was caused by the reduction in the number of phenotypes available to estimate breeding values.

Validation strategy 4 represented the situation where genotyped fish had no phenotypes in the dataset, which would eliminate the need to process genotyped fish. Predictive ability for residual carcass weight EBV decreased from 0.24 to 0.22, and of GEBV from 0.31 to 0.24. These results suggest that having genotypes for fish that are slaughtered for carcass weight recording is important and translates into the greatest benefit from genomic selection. Having phenotypes for genotyped individuals is important not only in aquaculture genomics, but in general livestock genomics. In a simulation study, Pszczola et al. [25] showed that the highest accuracies from genomic evaluation were obtained when animals from both reference (phenotyped) and evaluated (non phenotyped) populations were genotyped. Furthermore, Lourenco et al. [26] showed only one point increase in predictive ability in the genomic evaluation for calving ease in American Angus and related that to the small number of genotyped animals with records on difficult calving.

Although predictive ability decreased considerably when carcass records for genotyped fish were removed, ssGBLUP still outperformed traditional BLUP by about 9%. The improved performance of ssGBLUP in this situation is due to the fact that the ${\mathbf{H}}$ matrix connects genotyped animals without phenotypes to ungenotyped animals with phenotypes, if they are connected through the pedigree.

Overall, the use of genomic information improved the calculation of relationships among animals and allowed for a better estimation of Mendelian sampling, promoting an increase in predictive ability and allowing the use of within-family variation. Without genomic information, young full-sib fish (i.e., without phenotype or progeny) would have the same EBV for a trait, which equals to parent average [27].

**Fig. 1:**
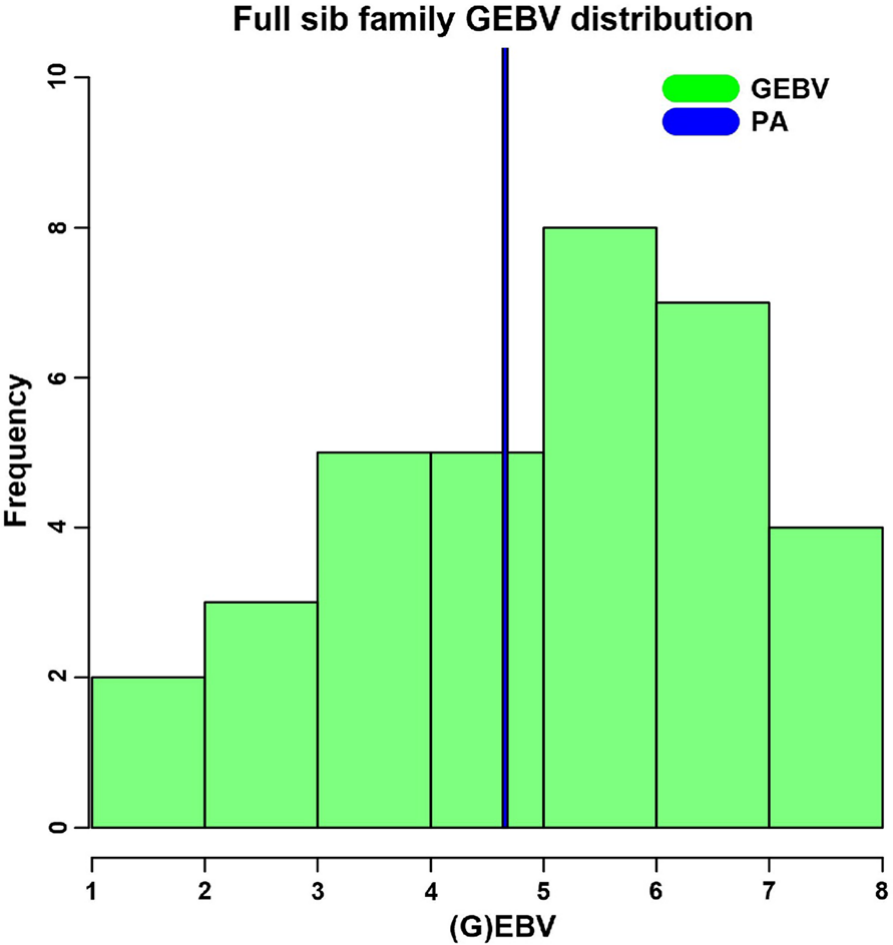
Distribution of genomic EBV for residual carcass weight (g) in a family of 34 young genotyped full-sibs

Lourenco et al. [28] showed that when an animal is genotyped but has no phenotype and progeny, the GEBV is composed of:

13 $${\text{GEBV}} = w_{ 1} {\text{PA + w}}_{ 2} {\text{GP}} - {\text{w}}_{ 3} {\text{PP,}}$$

where ${\text{PA}}$ is the parent average EBV for the animal, ${\text{GP}}$ is the portion of prediction due to the genomic information, coming from ${\mathbf{G}}$, and ${\text{PP}}$ is pedigree prediction that comes from ${\mathbf{A}}_{22}$; weights w1–w3 sum to 1. Quaas [29] described that the breeding value of an animal is the average of EBV from parents (${\text{PA}}$) plus a random term that takes into account the uncertainty about which 50% of the genes were passed to progeny (i.e., Mendelian sampling):

14 $${\text{EBV}} = 0 . 5 {\text{EBV}}_{\text{S}} + {0} . 5 {\text{EBV}}_{\text{D }} { + }\,{\varphi } ,$$

where ${\text{EBV}}_{\text{S}}$ is EBV from sire; ${\text{EBV}}_{\text{D}}$ is EBV from dam and ${\varphi }$ is the Mendelian sampling term. If the first portion of the formula corresponds to ${\text{PA}}$, ${\varphi }$ can be partially estimated by the genomic information present in ${\text{GP}}$, as shown in Eq. (13), because genomic data helps to estimate part of the uncertainty about which alleles and the proportion of alleles shared among individuals. Therefore, genotyped full-sibs that are selection candidates (i.e., young) have unique GEBV (not just ${\text{PA}}$) and the best candidates can be identified within families. Figure 1 shows the distribution of GEBV for a family of 34 full-sibs that had no phenotypes for residual carcass weight but were genotyped. Without genomic information, all 34 full-sibs had only PA, which is equal to 4.64 g. After including genomic information for all full-sibs, we observed a distribution ranging from 1.24 to 7.65. Use of GEBV would allow selection of fish within a family based on individual genetic merit for carcass weight, avoiding random selection of fish within a family based on BLUP ${\text{EBV}}_{\text{S}}$, which could result in selecting fish with in fact lower genetic merit.

The ability to identify selection candidates within a family that have higher genetic merit is a key benefit for a trait such as carcass weight in fish, which is not measured on selection candidates, and for quite large full-sib family sizes. Studies on other fish species such as Atlantic salmon [30–33] and rainbow trout [34, 35] have demonstrated increases in predictive ability or accuracy of GEBV compared to EBV, confirming the benefits of genomic selection for aquaculture species.

Tables 3 and 4 present EBV and GEBV inflation (${\text{b}}_{1}$) for harvest weight and residual carcass weight. In all validation scenarios, GEBV were less inflated or deflated compared to EBV, meaning that GEBV were closer in scale to the adjusted phenotypes. Updating variance components for each training dataset was beneficial for estimating inflation for both EBV and GEBV. The benefit comes from the fact that the variance components used to predict (G)EBV reflect the true state of the population after removing phenotypes for validation animals and therefore, less inflation is expected. Wiggans et al. [36] suggested that one way to reduce inflation of genomic evaluations of US cows would be to reduce heritability; this would be in line with a reduced additive genetic variation in recent generations. In our study, when variance components were re-estimated, the regression coefficients became closer to 1 and were the most beneficial for the cross-validation scenario for harvest weight, in which ${\text{b}}_{1} = 1$ for GEBV, meaning that GEBV and adjusted phenotypes had similar dispersion.

**Table 3:** Regression coefficients of adjusted phenotypes on EBV or GEBV for harvest weight

Validation strategy	Validation scenario	Same variance components		Updated variance components	
		BLUP	ssGBLUP	BLUP	ssGBLUP
1	Five fold cross-validation^a^	0.87^0.002^	0.92^0.002^	0.97^0.002^	1.00^0.002^
1	Ten fold cross-validation^a^	0.87^0.001^	0.92^0.001^	0.96^0.001^	1.00^0.001^
2	Full sib validation	0.94	0.98	1.05	1.04

^a^Average and standard error across five replicates

**Table 4:** Regression coefficients of adjusted phenotypes on EBV or GEBV for residual carcass weight

Validation strategy	Validation scenario	Same variance components		Updated variance components	
		BLUP	ssGBLUP	BLUP	ssGBLUP
1	Five fold cross-validation^a^	0.80^0.008^	0.91^0.007^	0.89^0.03^	0.94^0.007^
1	Ten fold cross-validation^a^	0.80^0.008^	0.92^0.005^	0.82^0.008^	0.95^0.005^
2	Full sib validation	0.83	1.08	0.85	1.10
3	Half of the full sibs with phenotypes	0.75	0.95	0.77	0.98
4	No phenotypes for all genotyped animals	0.76	0.87	0.79	0.90

^a^Average and standard error across five replicates

### Genome-wide association

**Fig. 2:**
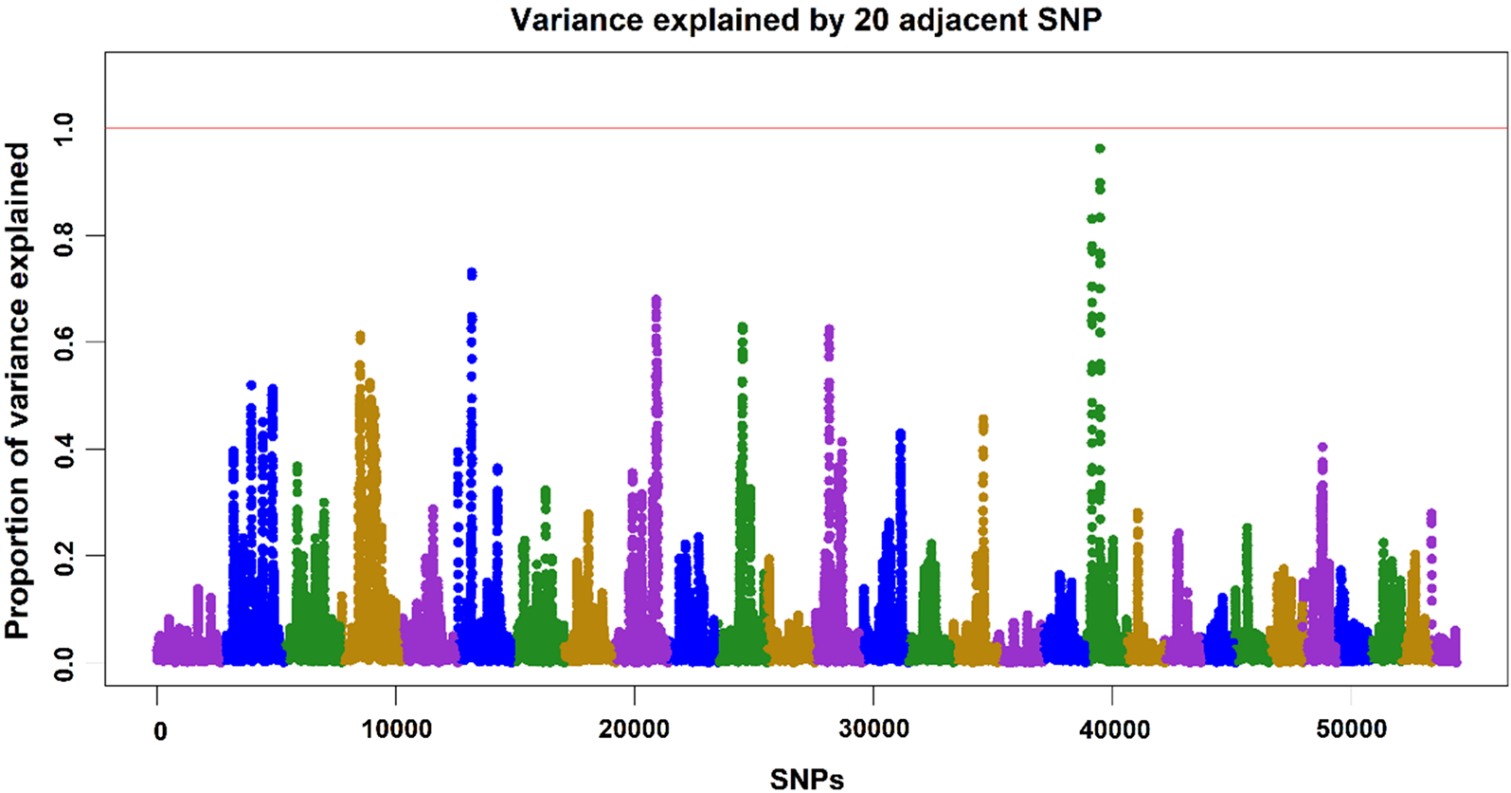
Manhattan plot for harvest weight in the 1st iteration of WssGBLUP, with the proportion of additive genetic variance explained by windows of 20 adjacent SNPs

**Fig. 3:**
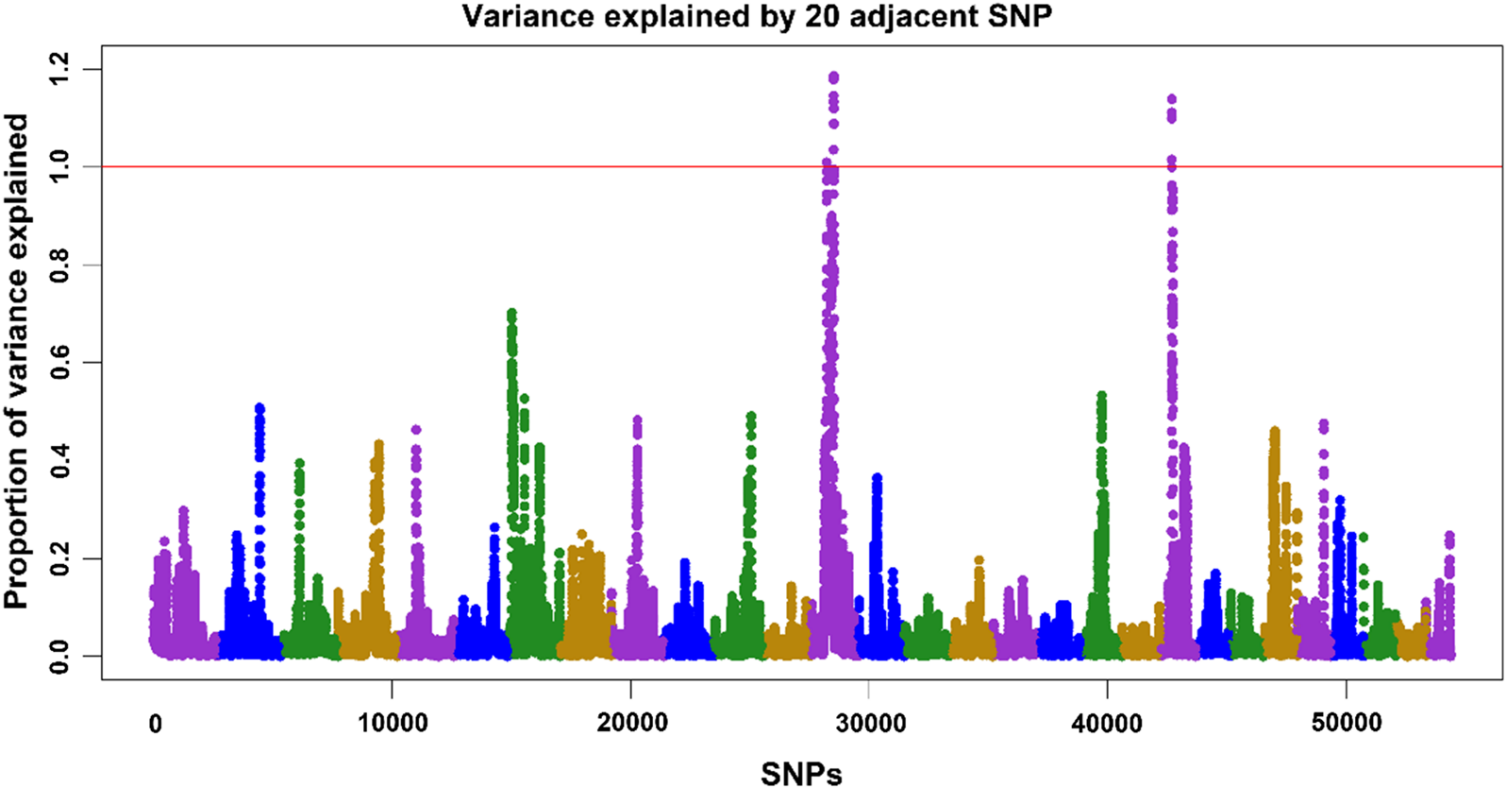
Manhattan plot for residual carcass weight in the 1st iteration of WssGBLUP, with the proportion of additive genetic variance explained by windows of 20 adjacent SNPs

Manhattan plots from the GWAS for harvest weight and residual carcass weight are shown in Figs. 2 and 3, respectively. The plots were drawn for the first iteration of WssGBLUP, because it had the greatest predictive ability and least inflation. In the first iteration, GEBV were computed assuming that all SNPs had the same weight. The GEBV were then back-solved to SNP effects and new weights were calculated and plotted as percentage of variance explained. Although predictive ability had to be computed based on the reduced dataset, the Manhattan plots were drawn based on the full dataset. The proportion of additive genetic variance explained by windows of 20 adjacent SNPs was up to 0.96% for harvest weight and up to 1.19% for residual carcass weight, which indicates that both traits are extremely polygenic. A single window explaining close to 1% of the additive genetic variation for harvest weight was located on chromosome 19, whereas, for residual carcass weight the top windows were located on chromosomes 13 and 21.

In an experimental population of less than 600 genotyped progeny of F1 males (channel x blue catfish) and channel catfish females, Li et al. [37] found a significant association between SNPs on chromosome 5 and body weight. These SNPs explained from 3.69 to 6.72% of the phenotypic variance for body weight. In a rainbow trout population from the National Center for Cool and Cold Water Aquaculture, Gonzalez-Pena et al. [38] found windows of 20 SNPs that explained more than 1% of the additive genetic variance for body weight at 10 and 13 months on chromosome 5, for fillet weight and yield on chromosome 9, and for carcass weight on chromosomes 9, 17, and 27. In our study, the windows that explained the top variance did not overlap with windows already described in the literature for the same species or trait.

The fact that top windows do not overlap even in populations from the same species has been described in the literature. Silva et al. [39] found very few overlapping genomic windows that explained more than 1% of the additive genetic variance for columnaris disease in two different rainbow trout populations. Fragomeni et al. [40] showed that, in a selected commercial broiler chicken population, the location of the windows with the largest effect was not consistent across different generations.

With a polygenic architecture and windows of SNPs explaining small proportions of the additive genetic variance, genomic selection for harvest weight and residual carcass weight in this catfish population is preferred over marker-assisted selection (MAS). Using MAS with such an architecture would not provide successful results given that only a small proportion of variance can be explained by individual SNPs.

Under a polygenic architecture, the use of Bayesian alphabet (e.g., BayesA, BayesB) and GBLUP-based methods that allow SNPs to explain a different proportion of variance (i.e., different SNP weightings; [20, 27]) may not help to increase the predictive ability or accuracy of GEBV. In fact, we observed that predictive ability for harvest weight and residual carcass weight did not change over the iterations of WssGBLUP when using non-linear A weights (results not shown). In addition, inflation slightly increased from iterations 1 to 3, reaching a plateau in later iterations (results not shown). When the best results for predictive ability and inflation are obtained in the first iteration of WssGBLUP, we can assume that using different weights is not beneficial, and, in this case, GEBV obtained from WssGBLUP are the same as in ssGBLUP. In a simulation study using linear SNP weights (i.e., $d_{j} = \hat{a}_{j}^{2} 2p_{j} \left( {1 - p_{j} } \right)$), Lourenco et al. [18] found that for more polygenic traits, decreases in accuracy or increases in inflation/deflation for WssGBLUP could be caused by the shrinkage of SNP weights for SNPs with smaller effects.

Although Manhattan plots were drawn based on the first iteration of WssGBLUP, the percentage of variance explained by SNPs did not change considerably over iterations. In fact, there was no change from iterations 2 to 5 for harvest weight and 3 to 5 for residual carcass weight. This possibly shows that non-linear A weights are not overestimated and they converge at some point. This convergence occurs because the formula contains a maximum limit for SNP weight. In an attempt to use the linear weights, we observed a constant increase in the proportion of variance explained (results not shown). This increase is due to the fact SNP weights keep changing over iterations without a limit for maximum change.

### Linkage disequilibrium and effective population size

**Fig. 4:**
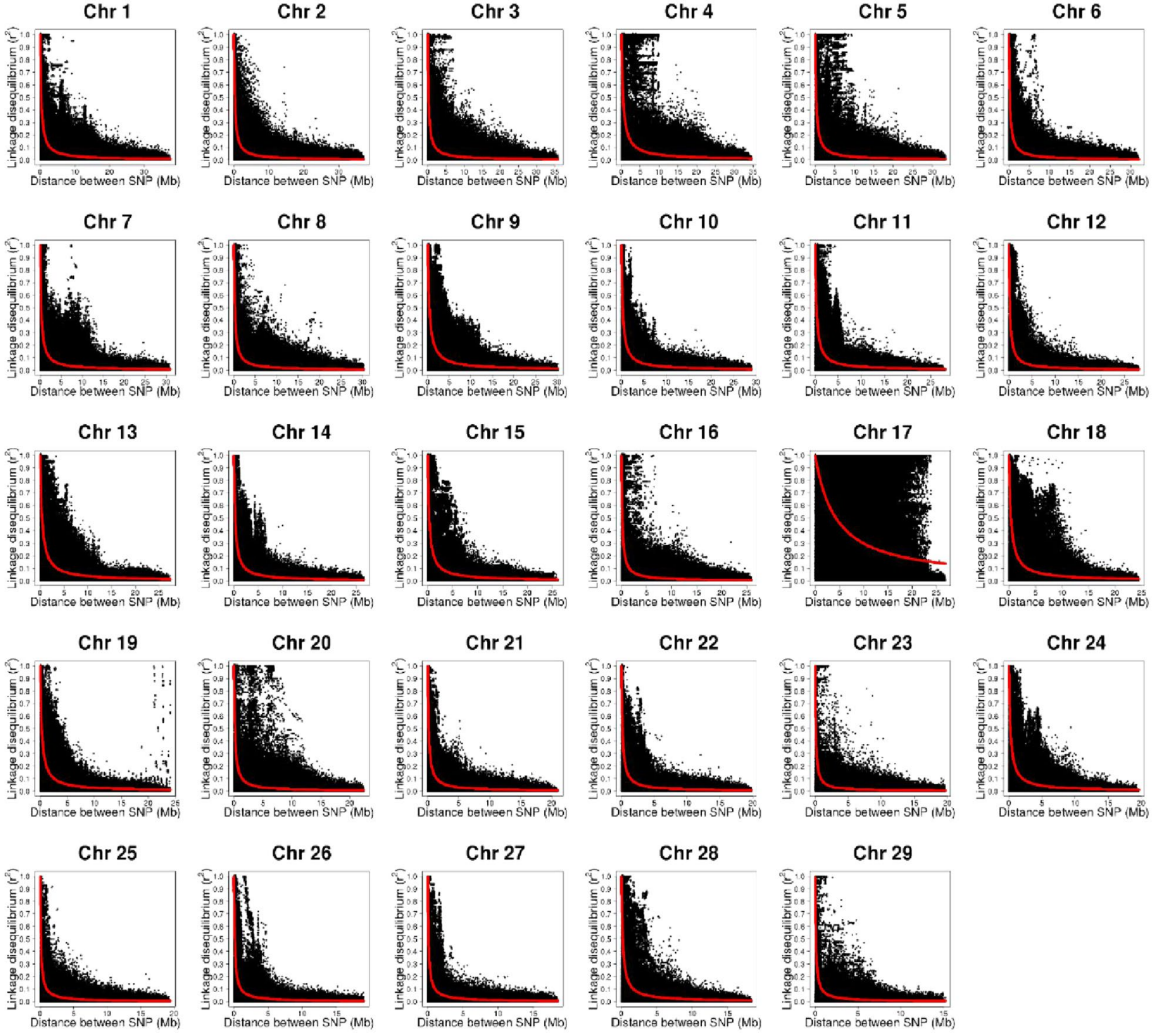
LD decay plots for 29 chromosomes

The overall whole-genome LD was 0.22 and ranged from a low value of 0.12 (chromosome 29) to a high value of 0.37 (chromosome 17). The LD was moderate even at long distances as shown in the LD decay plots in Fig. 4. There was a large, conservative LD block, which did not decay even at long distances (20 Mb) on chromosome 17, and a more in-depth investigation is needed to understand what might have caused this LD pattern.

The effective population size calculated based on LD and that based on inbreeding did not differ much, i.e. 27 and 28, respectively. Compared to livestock species, $N_{e}$ in catfish is relatively small. Pocrnic et al. [41] showed that $N_{e}$ for broiler chicken, swine, Angus cattle, Jersey, and Holstein cattle were 44, 32, 113, 101, and 149, respectively. In studies based on simulated populations, Pocrnic et al. [42] and Muir [7] associated $N_{e}$ with the dimensionality of the genomic information and showed higher accuracy of genomic predictions for smaller $N_{e}$. When $N_{e}$ is small, there are fewer and longer LD blocks, which can be well estimated even when the number of genotyped animals is less than 5000 [18]. In this way, the small $N_{e}$ in this catfish population may have contributed to the great increase in predictive ability even when only 2911 fish were genotyped (i.e., 8% of the population).

Considering the small effective population size and the long-range LD in this population, it might be possible to reduce the number of markers needed for genomic selection. Other studies have demonstrated similar accuracies when comparing low- and high-density SNP panels in salmonid species [23, 30, 32, 35]. Recently, Vallejo et al. [43] reported gains of accuracy (relative to traditional BLUP) of 88% for a 35 K SNP panel and 42% with a greatly reduced 200 SNP panel with ssGBLUP for bacterial cold water disease resistance in rainbow trout. The authors related the efficiency of the reduced SNP panel to the strong long-range LD in that rainbow trout population.

Reducing the density of markers in the panel would likely reduce genotyping costs and improve the cost efficiency of genomic selection in fish. More studies are necessary to investigate the overall cost and benefit of different SNP panel densities on implementation of genomic selection in this catfish population.

## Conclusions

Genomic information is beneficial for channel catfish breeding because it provides greater ability to predict future performance and reduces inflation of breeding values. For carcass traits, it is important to record carcass weight phenotypes on genotyped fish to obtain the largest advantage of genomic selection. Genomic information also allows the estimation of Mendelian sampling, enabling the identification of genetically superior individuals within families, which is not possible with pedigree information only. Genome-wide association suggests that harvest weight and residual carcass weight have a polygenic architecture, indicating that using many SNPs in a genome-wide selection approach would be superior to using fewer SNPs in a marker-assisted selection type of approach.

## References

[1] Vilsack T, Reilly JT. Census of aquaculture 2013. In: Agriculture USDo, editor. USDA, National Agricultural Statistics Service; 2013. p. 1–98.

[2] Meuwissen TH, Hayes BJ, Goddard ME (2001). Prediction of total genetic value using genome-wide dense marker maps. Genetics.

[3] Daetwyler HD, Villanueva B, Bijma P, Woolliams JA (2007). Inbreeding in genome-wide selection. J Anim Breed Genet.

[4] Aguilar I, Misztal I, Johnson DL, Legarra A, Tsuruta S, Lawlor TJ (2010). Hot topic: a unified approach to utilize phenotypic, full pedigree, and genomic information for genetic evaluation of Holstein final score. J Dairy Sci.

[5] Legarra A, Christensen OF, Aguilar I, Misztal I (2014). Single Step, a general approach for genomic selection. Livest Sci.

[6] Christensen OF, Lund MS (2010). Genomic prediction when some animals are not genotyped. Genet Sel Evol.

[7] Muir WM (2007). Comparison of genomic and traditional BLUP-estimated breeding value accuracy and selection response under alternative trait and genomic parameters. J Anim Breed Genet.

[8] Hayes BJ, Bowman PJ, Chamberlain AJ, Goddard ME (2009). Invited review: genomic selection in dairy cattle: Progress and challenges. J Dairy Sci.

[9] Waldbieser GC, Bosworth BG (2013). A standardized microsatellite marker panel for parentage and kinship analyses in channel catfish, *Ictalurus punctatus*. Anim Genet.

[10] Liu Z, Liu S, Yao J, Bao L, Zhang J, Li Y (2016). The channel catfish genome sequence provides insights into the evolution of scale formation in teleosts. Nat Commun.

[11] Li H. Aligning sequence reads, clone sequences and assembly contigs with BWA-MEM. .2013. arXiv preprint arXiv:13033997 [q-bio.GN].

[12] DePristo MA, Banks E, Poplin R, Garimella KV, Maguire JR, Hartl C (2011). A framework for variation discovery and genotyping using next-generation DNA sequencing data. Nat Genet.

[13] Misztal I, Tsuruta S, Lourenco DAL, Masuda Y, Aguilar I, Legarra A, et al. Manual for BLUPF90 family of programs. 2016.

[14] VanRaden PM (2008). Efficient methods to compute genomic predictions. J Dairy Sci.

[15] Reverter A, Golden B, Bourdon R, Brinks J (1994). Method R variance components procedure: application on the simple breeding value model. J Anim Sci.

[16] Wang H, Misztal I, Aguilar I, Legarra A, Muir WM (2012). Genome-wide association mapping including phenotypes from relatives without genotypes. Genet Res (Camb).

[17] Falconer DS, Mackay TFC (1996). Introduction to quantitative genetics.

[18] Lourenco DAL, Fragomeni BO, Bradford HL, Menezes IR, Ferraz JBS, Aguilar I (2017). Implications of SNP weighting on single-step genomic predictions for different reference population sizes. J Anim Breed Genet.

[19] Legarra A, Lourenco DA, Vitezica Z. Bases for genomic prediction 2018. http://genoweb.toulouse.inra.fr/~alegarra/GSIP.pdf. Accessed 10 Oct 2018.

[20] Zhang X, Lourenco D, Aguilar I, Legarra A, Misztal I (2016). Weighting strategies for single-step genomic BLUP: an iterative approach for accurate calculation of GEBV and GWAS. Front Genet.

[21] Sved JA (1971). Linkage disequilibrium and homozygosity of chromosome segments in finite populations. Theor Pop Biol.

[22] Saura M, Tenesa A, Woolliams JA, Fernández A, Villanueva B (2015). Evaluation of the linkage-disequilibrium method for the estimation of effective population size when generations overlap: an empirical case. BMC Genomics.

[23] Tsai HY, Hamilton A, Tinch AE, Guy DR, Bron JE, Taggart JB (2016). Genomic prediction of host resistance to sea lice in farmed Atlantic salmon populations. Genet Sel Evol.

[24] Meuwissen T, Hayes B, Goddard M (2016). Genomic selection: a paradigm shift in animal breeding. Anim Front.

[25] Pszczola M, Strabel T, van Arendonk JAM, Calus MPL (2012). The impact of genotyping different groups of animals on accuracy when moving from traditional to genomic selection. J Dairy Sci.

[26] Lourenco DA, Tsuruta S, Fragomeni BO, Masuda Y, Aguilar I, Legarra A (2015). Genetic evaluation using single-step genomic best linear unbiased predictor in American Angus. J Anim Sci.

[27] Daetwyler HD, Pong-Wong R, Villanueva B, Woolliams JA (2010). The impact of genetic architecture on genome-wide evaluation methods. Genetics.

[28] Lourenco DAL, Fragomeni BO, Tsuruta S, Aguilar I, Zumbach B, Hawken RJ (2015). Accuracy of estimated breeding values with genomic information on males, females, or both: an example on broiler chicken. Genet Sel Evol.

[29] Quaas RL (1988). Additive genetic model with groups and relationships. J Dairy Sci.

[30] Odegard J, Moen T, Santi N, Korsvoll SA, Kjoglum S, Meuwissen TH (2014). Genomic prediction in an admixed population of Atlantic salmon (*Salmo salar*). Front Genet.

[31] Tsai HY, Hamilton A, Tinch AE, Guy DR, Gharbi K, Stear MJ (2015). Genome wide association and genomic prediction for growth traits in juvenile farmed Atlantic salmon using a high density SNP array. BMC Genomics.

[32] Bangera R, Correa K, Lhorente JP, Figueroa R, Yáñez JM (2017). Genomic predictions can accelerate selection for resistance against *Piscirickettsia salmonis* in Atlantic salmon (*Salmo salar*). BMC Genomics.

[33] Correa K, Bangera R, Figueroa R, Lhorente JP, Yáñez JM (2017). The use of genomic information increases the accuracy of breeding value predictions for sea louse (*Caligus rogercresseyi*) resistance in Atlantic salmon (*Salmo salar*). Genet Sel Evol.

[34] Vallejo RL, Leeds TD, Gao G, Parsons JE, Martin KE, Evenhuis JP (2017). Genomic selection models double the accuracy of predicted breeding values for bacterial cold water disease resistance compared to a traditional pedigree-based model in rainbow trout aquaculture. Genet Sel Evol.

[35] Yoshida GM, Bangera R, Carvalheiro R, Correa K, Figueroa R, Lhorente JP (2018). Genomic prediction accuracy for resistance against *Piscirickettsia salmonis* in farmed rainbow trout. G3 (Bethesda).

[36] Wiggans GR, Cooper TA, VanRaden PM, Cole JB (2011). Technical note: adjustment of traditional cow evaluations to improve accuracy of genomic predictions. J Dairy Sci.

[37] Li N, Zhou T, Geng X, Jin Y, Wang X, Liu S (2018). Identification of novel genes significantly affecting growth in catfish through GWAS analysis. Mol Genet Genomics.

[38] Gonzalez-Pena D, Gao G, Baranski M, Moen T, Cleveland BM, Kenney PB (2016). Genome-wide association study for identifying loci that affect fillet yield, carcass, and body weight traits in rainbow trout (*Oncorhynchus mykiss*). Front Genet.

[39] Silva RMO, Evenhuis JP, Valejo R, Gao G, Martin KE, Misztal I, et al. GWAS for detecting QTL associated with Columnaris disease in two rainbow trout breeding populations. In: Proceedings of the plant anim genome XXXVI conference: 13–17 January 2018; San Diego. 2018.

[40] Fragomeni Bde O, Misztal I, Lourenco DL, Aguilar I, Okimoto R, Muir WM (2014). Changes in variance explained by top SNP windows over generations for three traits in broiler chicken. Front Genet.

[41] Pocrnic I, Lourenco DAL, Masuda Y, Misztal I (2016). Dimensionality of genomic information and performance of the Algorithm for Proven and Young for different livestock species. Genet Sel Evol.

[42] Pocrnic I, Lourenco DAL, Masuda Y, Legarra A, Misztal I (2016). The dimensionality of genomic information and its effect on genomic prediction. Genetics.

[43] Vallejo RL, Silva RMO, Evenhuis JP, Gao G, Liu S, Parsons JE (2018). Accurate genomic predictions for BCWD resistance in rainbow trout are achieved using low-density SNP panels: Evidence that long-range LD is a major contributing factor. J Anim Breed Genet.

